# Mitoxantrone Therapy for Acute Posterior Multifocal Placoid Pigment Epitheliopathy with Cerebral Vasculitis

**DOI:** 10.1155/2009/481512

**Published:** 2009-05-19

**Authors:** Hélène Massé, Jean-Laurent Guyomard, Dominique Baudet, Jean-François Pinel, Gilles Edan, Jean-François Charlin

**Affiliations:** ^1^Service d'ophtalmologie, CHU Pontchaillou, rue Henri Le Guilloux, 35000 Rennes, France; ^2^Service de neurologie, CHU Pontchaillou, rue Henri Le Guilloux, 35000 Rennes, France

## Abstract

*Purpose*. To report favorable outcome of a case of acute posterior multifocal placoid pigment epitheliopathy (APMPPE) associated with cerebral vasculitis after treatment with immunosuppressive therapy by mitoxantrone. *Design*. Single case report. *Method*. A 22-year-old man presented with acute isolated bilateral loss of vision revealing APMPPE. Corticosteroid therapy was initiated and visual acuity gradually improved. Seventeen days later, visual function deteriorated again, associated with flu-like syndrome and severe headaches. A relapse of APMPPE was diagnosed, complicated with lymphocytic meningitis and cerebral ischemia. Intravenous therapy with mitoxantrone was performed in combination with methylprednisolone. *Results*. Headaches disappeared in a few days whereas visual acuity gradually improved and stabilized at 20/40 in the right eye and 20/32 in the left eye. No adverse event was observed. Clinical improvement was confirmed by magnetic resonance imaging. *Conclusion*. Cerebral vasculitis is the most severe complication of the extraocular manifestations of APMPEE. This diagnosis should be evoked when severe headaches or behavior disorder are associated with APMPEE.

Acute posterior multifocal placoid pigment epitheliopathy (APMPPE) was first described by Gass [1] as multiple creamy white lesions at the level of the retinal pigment epithelium with rapid loss of central vision and spontaneous recovery within two to three weeks. The disorder invariably affects both eyes with preponderance for young adults. Choroidal vasculitis has been proposed as the underlying pathological process [2]. Since the initial report, systemic abnormalities have been described in association with APMPPE including polyarteritis and systemic necrotizing vasculitis with Wegener's granulomatosis [3]. Cerebral vasculitis is rare but potentially and rapidly fatal [4]. We report a case of APMPPE-associated cerebral vasculitis with favorable outcome after treatment with immunosuppressive therapy by mitoxantrone.

A 22-year-old man presented with sudden isolated bilateral visual loss. He had no previous medical history.The initial visual acuity was limited to counting fingers at a distance of 50 cm in both eyes. Fundus examination disclosed a mild vitreous haze and multiple yellow-white placoid lesions involving the retinal pigment epithelium in both posterior poles. Characteristic hypofluorescence in the early phase of the angiogram followed by late hyperfluorescence of the lesions confirmed the diagnosis of APMPPE (Figure 1(a) and  1(b)). Intravenous methylprednisolone was initiated, and visual acuity gradually improved. Seventeen days later, the patient experienced recurrence of visual loss associated with flu-like syndrome and severe headaches. Ophthalmologic examination did not reveal obvious new retinal lesion. Cerebro-spinal fluid analysis showed lymphocytic pleiocytosis. Magnetic resonance imaging (MRI) revealed bilateral parieto-occipital infarcts (Figures 2  and 3). Alternative diagnoses were excluded by extensive laboratory and vascular investigations. In collaboration with the neurologists, three intraveinous infusions of 20 mg mitoxantrone associated once with 1 g methylprednisolone and anticoagulation by antivitamin K were performed during a nine-month period.

 Headaches disappeared in a few days while visual acuity gradually improved over 3 months stabilizing at 20/40 in the right eye and 20/32 in the left eye. Clinical improvement was confirmed by MRI showing regression of occipital lesions. Cerebrospinal fluid analysis was normalized. Treatment was performed under close clinical, haematological, and echocardiographical monitoring. No adverse effect was detected.

Systemic vasculitis associated with APMPPE has been previously described. These associations provide support for the choroid as being primarily involved by a diffuse vasculitic process that interrupts choroidal perfusion and causes the characteristic fundus findings in APMPPE. 

Because of the potential severity of cerebral involvement associated with APMPPE, immunosuppressive therapy, such as cyclophosphamide, is usually considered. However, side effects of this drug are significant, especially in young adults, in particular transitory or definitive amenorrhoea and azoospermia.

Mitoxantrone, a synthetic anthracedione, is an established cytotoxic, antineoplastic agent, now approved in the treatment of multiple sclerosis [5] for its presumed mechanism of immunosuppression. The drug was generally well tolerated at the recommended dosage [6], although potential cardiotoxicity limits the total cumulative dose and requires strict echocardiographical monitoring. 

To the best of our knowledge, there is no mention in literature of previous report relating prescription of mitoxantrone in this indication. In cases of diffuse cerebral vasculitis, this treatment could be considered as a therapeutic alternative.

Cerebral vasculitis is the most severe complication of the extraocular manifestations of APMPEE. Although rare, this complication can be fatal. Hence the diagnosis should be evoked without delay when severe headaches or behavior disorders are associated with APMPPE.

## Figures and Tables

**Figure 1:**
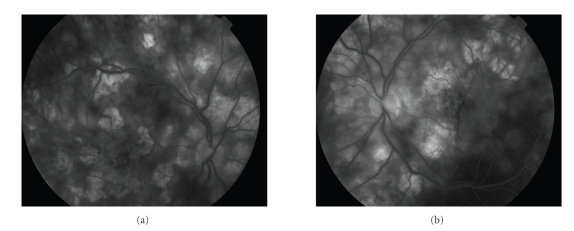
Fluorescein angiography of (a) right and (b) left eye demonstrates a diffuse staining of the placoid lesions in the late frames.

**Figure 2:**
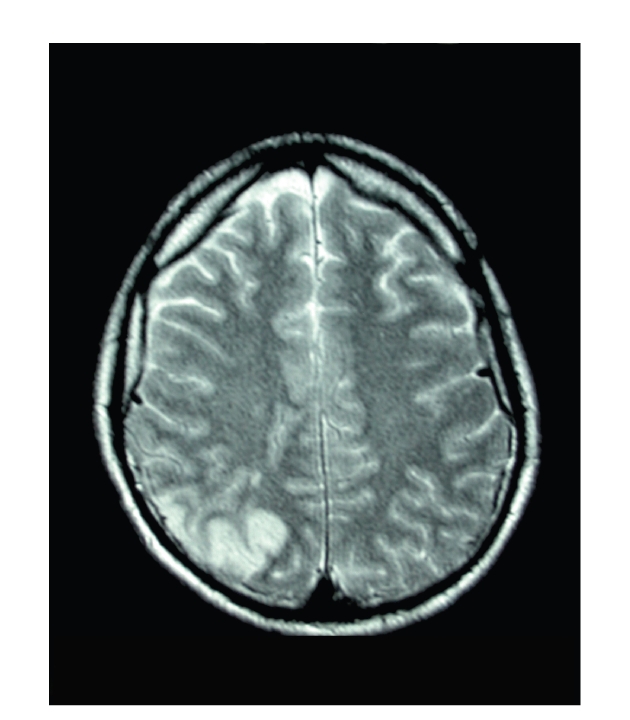
T2-weighted MRI showing bilateral hyperintense parieto-occipital lesions.

**Figure 3:**
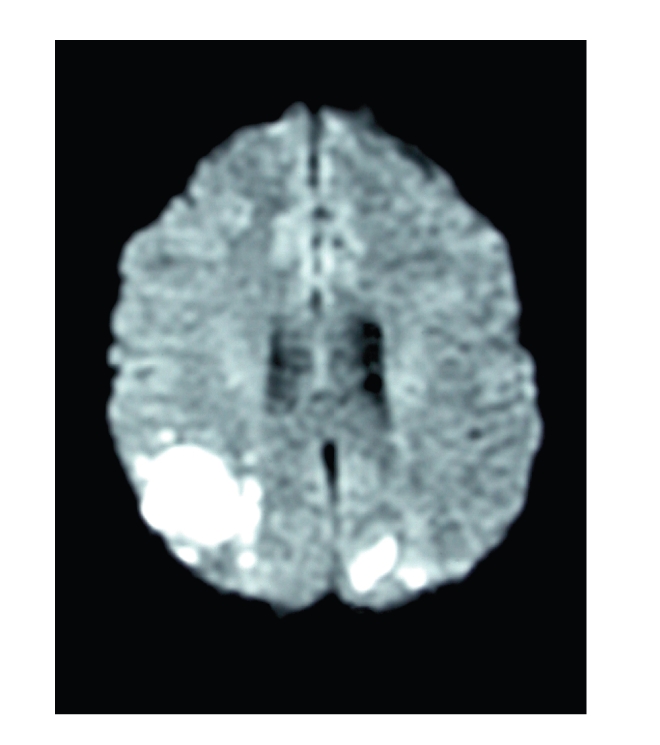
Diffusion tensor MRI emphasizing bilateral ischemia of parieto-occipital territories.
